# Living Donor Liver Transplantation for Vasoactive Intestinal Peptide-Secreting Tumors with Watery Diarrhea, Hypokalemia, and Achlorhydria Syndrome Resulting from Transformation of a Nonfunctional Pancreatic Neuroendocrine Tumor with Multiple Liver Metastases: A Case Report

**DOI:** 10.70352/scrj.cr.26-0465

**Published:** 2026-10-10

**Authors:** Satoshi Amano, Hiroyuki Nitta, Akira Umemura, Daiki Takeda, Taro Ando, Toma Kawashima, Koji Kikuchi, Taku Kimura, Yoshiyuki Ihara, Ayaka Sato, Naoki Yanagawa, Akira Sasaki

**Affiliations:** 1 Department of Surgery, School of Medicine, Iwate Medical University; 2 Department of Molecular Diagnostic Pathology, School of Medicine, Iwate Medical University

**Keywords:** liver transplantation, PNEN, VIPoma, transformation, WDHA syndrome

## Abstract

**INTRODUCTION:**

Pancreatic neuroendocrine neoplasms (PNENs) are a rare subtype of neuroendocrine neoplasms (NENs), some of which cause characteristic clinical syndromes due to hormone hypersecretion. Vasoactive intestinal peptide-secreting tumors (VIPomas) produce vasoactive intestinal peptide (VIP) and are associated with watery diarrhea, hypokalemia, and achlorhydria (WDHA) syndrome, often leading to severe treatment-refractory symptoms. Although liver transplantation (LT) is regarded as a promising option for unresectable, liver-limited NENs, studies focusing on VIPoma remain limited, as do reports of transformation from nonfunctional pancreatic NENs (PNENs) to VIPoma. Furthermore, to the best of our knowledge, no cases have described progression to LT after such a transformation. Therefore, we report this case.

**CASE PRESENTATION:**

A 65-year-old woman with nonfunctional PNEN G2 and multiple liver metastases underwent multimodal treatment including a multi-kinase inhibitor, somatostatin analogs, a mechanistic target of rapamycin (mTOR) inhibitors, and peptide receptor radionuclide therapy (PRRT). During treatment, the tumor acquired VIP-secreting capacity and transformed into a VIPoma, resulting in WDHA syndrome characterized by intractable watery diarrhea, severe electrolyte imbalance, and metabolic acidosis. Because neither conservative management nor debulking surgery achieved adequate symptom control, and no extrahepatic disease was identified, living donor LT (LDLT) was performed. After transplantation, the diarrhea resolved promptly, and no recurrence has been observed 1 year post-transplantation.

**CONCLUSIONS:**

We report a rare case in which a nonfunctional PNEN transformed into a VIPoma during the clinical course, leading to severe WDHA syndrome. LDLT for liver-limited disease may represent an effective therapeutic option for both tumor control and symptom relief in carefully selected patients.

## Abbreviations

EUS-FNA
: endoscopic US-guided fine-needle aspiration
LDLT
: living donor liver transplantation
LT
: liver transplantation
mTOR
: mechanistic target of rapamycin
NEN
: neuroendocrine neoplasm
PNEN
: pancreatic neuroendocrine neoplasm
PPPD
: pylorus-preserving pancreaticoduodenectomy
PRRT
: peptide receptor radionuclide therapy
VIP
: vasoactive intestinal peptide
VIPoma
: vasoactive intestinal peptide-secreting tumor
WDHA
: watery diarrhea, hypokalemia, and achlorhydria

## INTRODUCTION

PNENs are relatively rare tumors; however, their incidence has increased recently owing to advances in imaging and pathological diagnostics.^1–4)^ PNENs are classified as functional or nonfunctional according to hormone secretion, and their clinical presentation and treatment strategies vary widely depending on functional status, proliferative activity, and metastatic spread.

VIPomas are functional PNENs characterized by excessive secretion of VIP. VIPomas are extremely rare neoplasms; according to the 2023 World Health Organization (WHO) classification, they account for only 0.6%–1.5% of all PNENs and approximately 2%–6% of all functional PNENs.^5)^ Although 80%–90% of VIPomas are of pancreatic origin, the majority (60%–80%) are identified as metastatic malignant diseases, typically involving the liver, at the time of diagnosis. Their prognosis depends heavily on the presence of distant metastasis and the feasibility of surgical resection.^1,6)^

VIPomas cause WDHA, which is referred to as WDHA syndrome.^7)^ In severe cases, profound dehydration and electrolyte disturbances may lead to life-threatening conditions, requiring prompt and appropriate therapeutic intervention.

For unresectable PNENs with liver metastases, multimodal therapies, including somatostatin analogs, molecular-targeted agents, and PRRT, are widely used. In contrast, LT for liver-limited disease has been proposed as a potentially curative strategy in carefully selected patients. However, reports of LT specifically for VIPoma remain extremely limited, perhaps due to both the rarity of the disease and its frequent presentation as advanced or metastatic at diagnosis.^8)^ Transformation of nonfunctional PNENs into functioning tumors through secondary hormone secretion is rare, and transformation into VIPomas is extremely rare.^9)^

Herein, we describe a rare case of nonfunctional PNEN with multiple liver metastases that transformed into VIPoma during treatment, leading to severe WDHA syndrome, and that was successfully treated with LDLT. This case underscores both the phenomenon of tumor transformation and the potential role of LDLT in managing advanced VIPomas.

## CASE PRESENTATION

A 65-year-old woman was referred to our hospital at initial presentation after a pancreatic tumor was detected on abdominal ultrasonography screening. Contrast-enhanced CT demonstrated a 40-mm tumor in the pancreatic head accompanied by 8 bilobar liver metastases ranging from 5 to 13 mm in diameter (**Fig. 1**). EUS-FNA and liver biopsy confirmed the diagnosis of PNEN G2 with a Ki-67 index of 20% (**Fig. 2**).

**Fig. 1:**
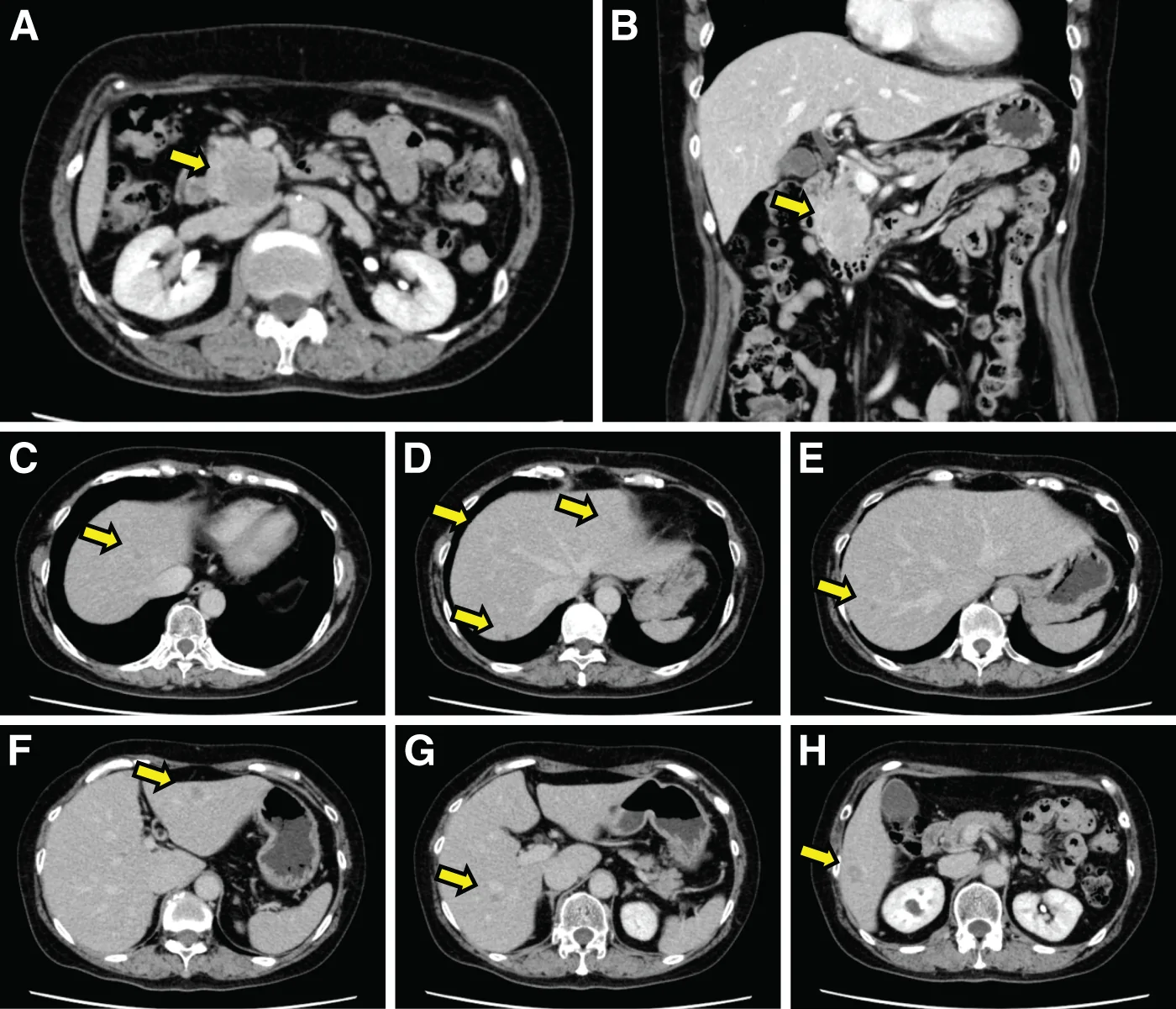
Contrast-enhanced CT findings at initial presentation. Axial and coronal contrast-enhanced CT images. A 40-mm tumor is observed in the pancreatic head (**A**, **B**). Multiple bilobar metastatic lesions are also observed in the liver (indicated by arrows; **C**–**H**).

**Fig. 2:**
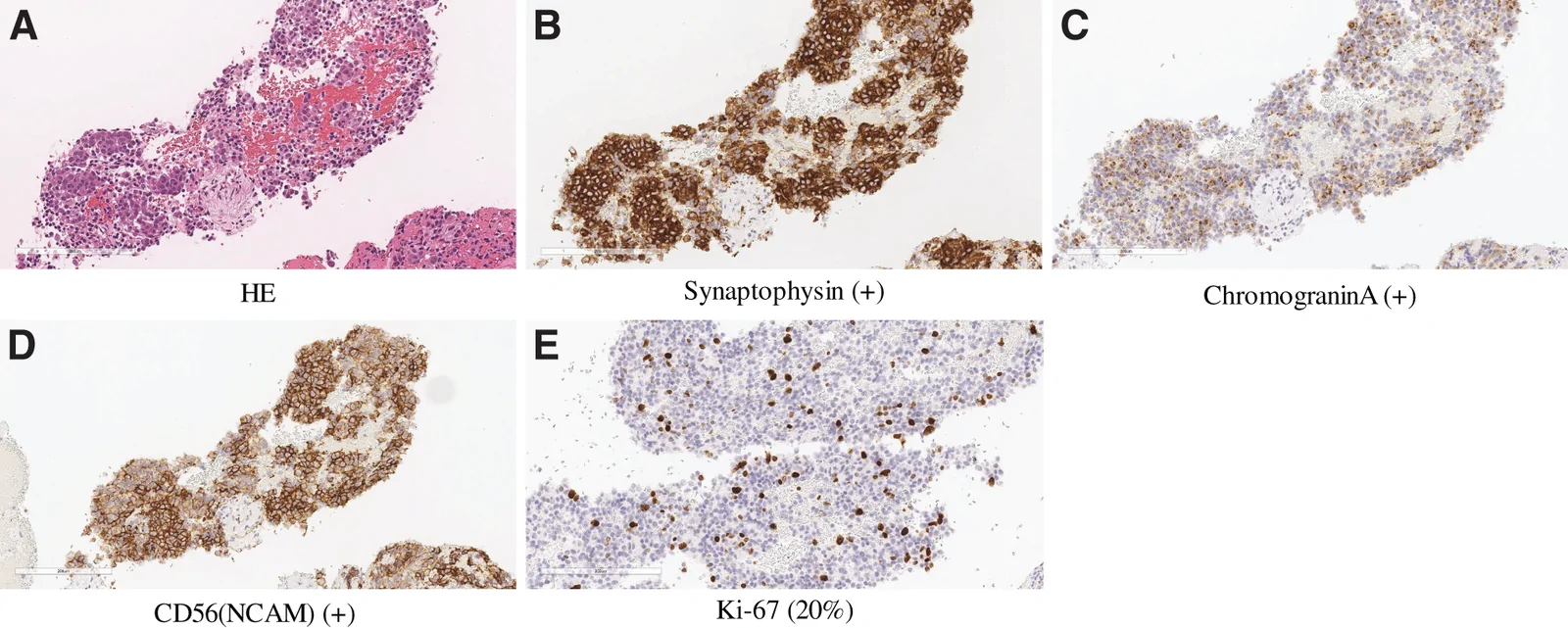
Histopathological findings of biopsy specimens from the pancreatic tumor and liver metastases at initial presentation. Biopsy specimens of the pancreatic tumor. Hematoxylin and eosin staining (**A**) demonstrated a trabecular growth pattern of tumor cells. On immunohistochemical staining, the tumor cells were positive for AE1/AE3, synaptophysin (**B**), chromogranin A (**C**), and CD56 (**D**), indicating neuroendocrine differentiation. The Ki-67 labeling index was approximately 20% (**E**), and the tumor was diagnosed as a NEN G2. Similar histopathological and immunohistochemical findings were identified in the liver biopsy, indicating metastatic disease. CD56, cluster of differentiation 56; HE, hematoxylin and eosin staining; NEN, neuroendocrine neoplasm

Treatment with sunitinib was initiated. The liver metastases disappeared on imaging within 6 months and remained undetectable, with no regrowth or new lesions, for the subsequent 12 months, while the primary tumor showed a shrinking trend. After 18 months of therapy, as the initially unresectable, bilobar disease had responded markedly and become resectable, conversion surgery with curative intent was planned (**Fig. 3**). The patient underwent PPPD. However, intraoperative inspection showed nodular lesions on the liver surface, and a partial hepatectomy was performed. Histopathological examination demonstrated PNEN G2 in the primary lesion and metastatic disease in the liver (**Fig. 4**). Adjuvant sunitinib therapy was subsequently resumed.

**Fig. 3:**
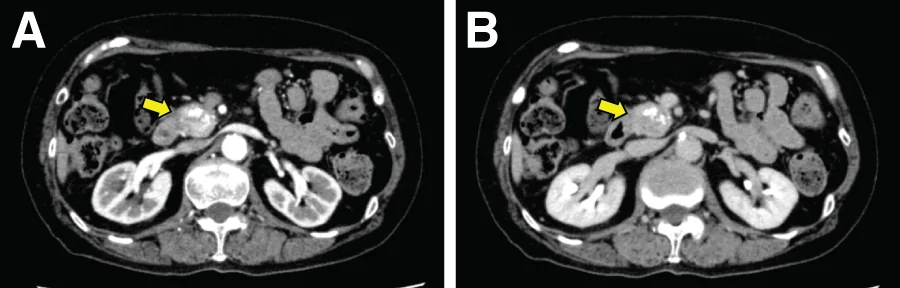
Contrast-enhanced CT before PPPD. The arterial phase (**A**) and portal venous phase (**B**) are presented. A tumor measuring approximately 30 mm in diameter is observed in the pancreatic head. Multiple liver metastases remained reduced in size, with no evidence of new lesions, lymph node metastases, or distant metastases. PPPD, pylorus-preserving pancreaticoduodenectomy

**Fig. 4:**
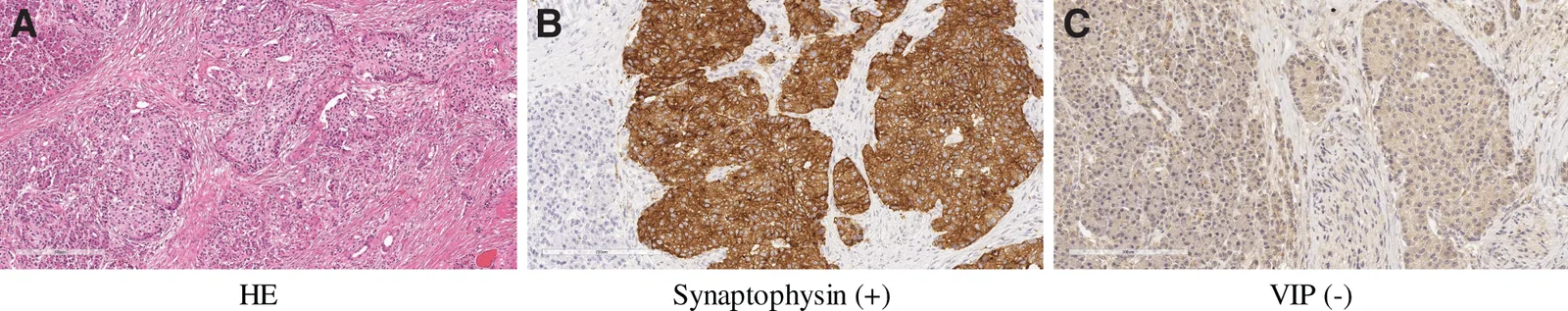
Histopathological findings of the pancreatic tumor and liver lesions after PPPD. The resected pancreatic tumor specimen after PPPD. Hematoxylin and eosin staining (**A**) and immunohistochemical analyses, including synaptophysin positivity (**B**), showed findings similar to those of the preoperative biopsy, and the tumor was diagnosed as a NEN G2. The liver lesions demonstrated similar histopathological features. Lymph node metastases were identified in 3 of 19 nodes. According to the Union for International Cancer Control (UICC) 8th edition, the tumor was classified as T2N1M1, Stage IV. Immunohistochemical staining for VIP did not show any apparent positivity (**C**). NEN, neuroendocrine neoplasm; PPPD, pylorus-preserving pancreaticoduodenectomy

Four months after surgery, contrast-enhanced CT demonstrated recurrent multiple liver metastases. Treatment with a somatostatin analog and everolimus was initiated; however, the disease continued to progress. Somatostatin receptor scintigraphy revealed abnormal uptake in the liver, and PRRT with a radiolabeled somatostatin analog (^177^Lu-DOTATATE) was planned 15 months postoperatively.

During PRRT, the patient developed worsening diarrhea and severe dehydration, necessitating hospitalization. Specifically, she experienced massive, intractable watery diarrhea ranging from 7000 to 10000 mL per day, accompanied by severe hypokalemia, electrolyte imbalance, and metabolic acidosis. Because serum VIP measurement is not currently available as a clinical assay in Japan, serum VIP was not measured, and the diagnosis of VIPoma was therefore established pathologically. Immunohistochemical analysis of liver biopsy specimens demonstrated VIP positivity, indicating transformation of a metastatic nonfunctional PNEN into a VIP-producing tumor through secondary hormone secretion, which led to WDHA syndrome (**Fig. 5**).

**Fig. 5:**
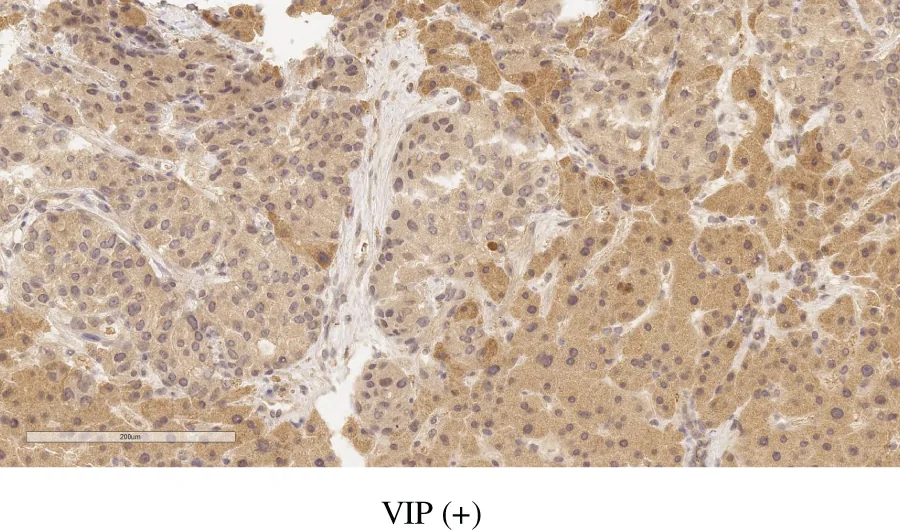
Biopsy of liver metastases obtained before LT. Immunohistochemical staining demonstrated VIP positivity in tumor cells, indicating functional transformation associated with secondary hormone secretion. LT, liver transplantation; VIP, vasoactive intestinal peptide

Despite intensive care, including fluid resuscitation, electrolyte correction, and octreotide administration in the ICU, the symptoms were uncontrollable. The persistence of the diarrhea, which had developed during PRRT, clinically demonstrated that liver metastases were the primary source of VIP production, thereby justifying LT as a necessary rescue therapy.

After 6 months of observation without evidence of extrahepatic metastasis (**Figs. 6**, **7**), LDLT using an ABO-compatible right lobe graft was performed 24 months after the initial surgery, at which time the patient was 69 years old. Extensive dense adhesions were identified intraoperatively in the upper abdomen, caused by the previous PPPD, which were meticulously dissected. After thorough abdominal exploration confirmed the absence of unresectable factors, including peritoneal dissemination or lymph node metastasis, a total hepatectomy was completed in the standard fashion. Regarding vascular reconstruction, the graft’s right hepatic vein was anastomosed to the recipient’s inferior vena cava. The graft’s right portal vein was anastomosed to the recipient’s right portal vein, and the graft’s right hepatic artery was anastomosed to the recipient’s middle hepatic artery. For biliary reconstruction, the previous biliary–enteric anastomosis was divided, and biliary–enteric re-anastomosis was performed using the preserved jejunal limb. The total operative time was 720 min, with an estimated blood loss of 4217 mL.

**Fig. 6:**
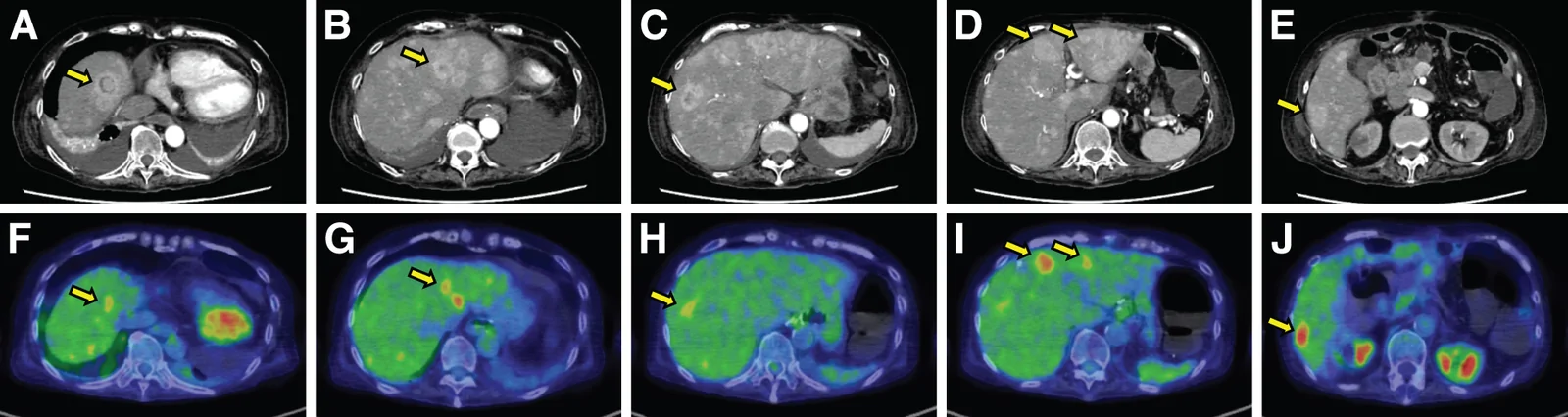
Imaging findings of the multiple liver metastases before LT. Contrast-enhanced CT in the arterial phase (**A**–**E**) and PET-CT (**F**–**J**) are shown; each column corresponds to the same anatomical level. Contrast-enhanced CT demonstrates innumerable liver metastases distributed throughout both hepatic lobes, and the arrows indicate lesions showing corresponding increased uptake on PET-CT. No apparent extrahepatic metastases were identified. LT, liver transplantation

**Fig. 7:**
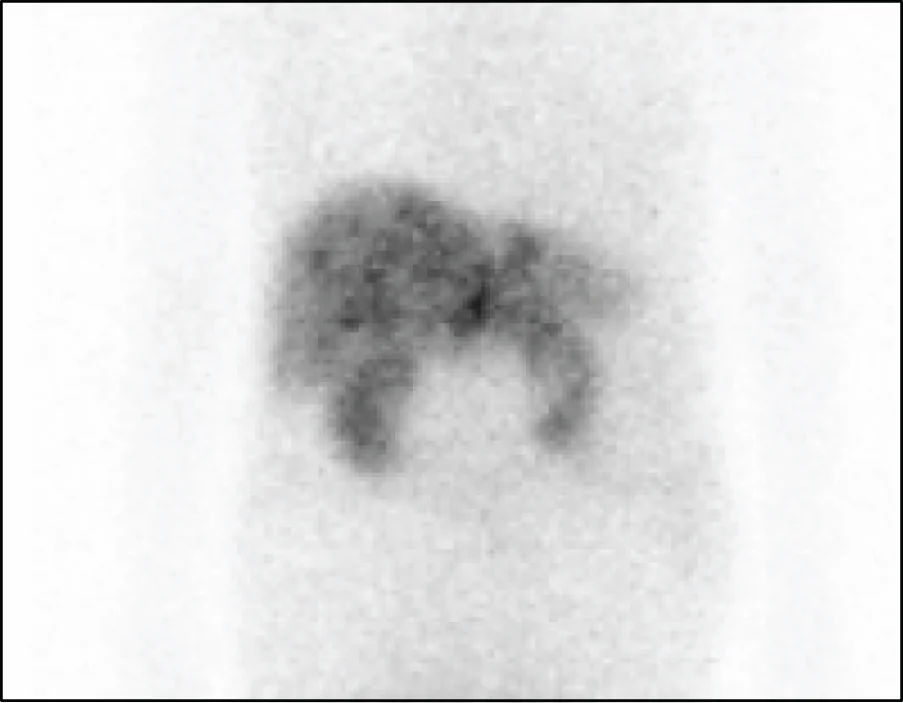
Somatostatin receptor scintigraphy before LT. Multiple areas of increased uptake are observed throughout the liver, corresponding to the multiple liver metastases. No abnormal uptake suggestive of extrahepatic metastases was identified. LT, liver transplantation

The severe diarrhea resolved immediately after transplantation. Postoperative immunosuppression consisted of tacrolimus and prednisolone according to our standard protocol. In addition, everolimus was incorporated into the regimen, taking advantage of its dual immunosuppressive and antitumor activity as an mTOR inhibitor against the neuroendocrine tumor. Although prolonged hospitalization was required for rehabilitation and infection management, no rejection occurred. One year after transplantation, the patient remained free of recurrent symptoms and tumor recurrence.

## DISCUSSION

We experienced an extremely rare case of a nonfunctional PNEN transforming into a VIPoma during the clinical course, resulting in severe WDHA syndrome. Despite multidisciplinary treatment, including PRRT, the patient’s life-threatening symptoms were controlled only after LDLT. To the best of our knowledge, this is the first report describing the phenotypic transformation of a nonfunctional PNEN into a VIPoma successfully treated with LDLT. This case underscores the importance of monitoring functional changes in advanced PNENs and indicates that LT is a definitive therapeutic option for refractory WDHA syndrome.

As mentioned above, PNENs are rare subtypes of NENs, with an annual incidence of approximately 1–2 cases per 100000 individuals, although recent reports suggest a rising incidence.^1–4)^ VIPomas are particularly rare functional PNENs characterized by excessive secretion of VIP. VIP, first identified in 1966, is a gastrointestinal peptide hormone encoded by a gene on chromosome 6 and has a short plasma half-life (approximately 2 min).^10–12)^ By stimulating intracellular cyclic adenosine monophosphate (cAMP), VIP induces intestinal water and electrolyte secretion, inhibits gastric acid secretion, and exerts various effects on the cardiovascular, respiratory, immune, and nervous systems (**Table 1**).^1,12,13)^ Excessive VIP secretion results in WDHA syndrome, a hallmark clinical feature of VIPoma. In cases with liver metastases, hormone-related symptoms may become more clinically apparent due to increased systemic hormone release and decreased hepatic metabolism.^7)^

**Table 1:** Systemic physiological effects of VIP across organ systems

Gastrointestinal effect	Vaso-cardiac effect	
• Increases insulin and glucagon secretion • Increases intestinal luminal fluid and electrolyte secretion • Increases epithelial cell proliferation • Increases pancreatic enzyme release • Decreases intestinal paracellular permeability • Decreases absorption of luminal intestinal fluid	• Increases cardiac contractility • Increases cardiac output • Vasodilatation • Lowers blood pressure • Arrhythmia	
Neuronal effect	Immune system effect	Respiratory effect
• Relaxes smooth muscles • Regulates circadian rhythm • Promotes brain development and enhances neuronal survival	• Anti-inflammatory effect • Promotes differentiation of T-cell • Promotes deactivation of macrophages	• Promotes bronchodilatation

VIP, vasoactive intestinal peptide

LT has emerged as a potential treatment option for unresectable, liver-limited NENs. Several transplant programs have proposed selection criteria, among which the Milan-neuroendocrine tumor (NET) criteria and United Network for Organ Sharing guidelines are widely accepted.^14–16)^ With strict patient selection, favorable long-term outcomes have been reported. Since the first report in 1990, only 9 cases of LT for VIPoma have been described in the literature (**Table 2**).^6,17–22)^ In almost all reported cases, VIPomas originate in the pancreas, and WDHA syndrome is an inevitable manifestation when liver metastases are present. LT has consistently achieved symptomatic relief in nearly all of these cases. Among NEN subtypes, VIPomas seem associated with relatively favorable outcomes following transplantation, with reported recurrence-free survival rates of 84%, 47%, and 47% at 1, 3, and 5 years, respectively.^8)^

**Table 2:** Reported cases of VIPoma treated with LT

Authors	Sex	Age	Primary location	WDHA syndrome	Symptomatic improvement after LT	Time to recurrence (month)	Survival (month)
Bramley et al. 1990^17)^	F	41	NA	Yes	Yes	-	12
Hengst et al. 1998^18)^	F	46	Pancreas	Yes	Yes	-	NA
Lang et al. 1999^19)^	F	47	Pancreas	Yes	Yes	48	117
	F	43	Pancreas	Yes	Yes	11	35
Florman et al. 2004^20)^	M	52	Pancreas	Yes	Yes	-	123
	M	59	Pancreas	Yes	NA	NA	0
van Vilsteren et al. 2006^21)^	F	62	Pancreas	Yes	Yes	-	73
Johnston et al. 2010^6)^	M	48	Pancreas	NA	Yes	-	108
Andreassen et al. 2024^22)^	F	40	Pancreas	Yes	Yes	15	18
Our case 2026	F	69	Pancreas	Yes	Yes	-	12

For our case, the listed age (69 years) is the age at LT; the patient was 65 years old at initial presentation.

‐, no recurrence; F, female; LT, liver transplantation; M, male; NA, not available; VIPoma, vasoactive intestinal peptide-secreting tumor; WDHA, watery diarrhea, hypokalemia, and achlorhydria

Tumor transformation and secondary hormone secretion in PNENs are most commonly reported in cases involving transformation into insulinomas.^23)^ Although advanced disease may exhibit secondary secretion of multiple hormones, transformation into VIPomas remains extremely rare.^9,24)^

In the present case, the metastatic PNEN acquired VIP-secreting capability during treatment, leading to refractory WDHA syndrome. Despite aggressive multimodal therapy, including somatostatin analogs, molecular-targeted agents, and PRRT, successful tumor control and symptom relief were not achieved. The patient’s QOL declined significantly because of uncontrollable diarrhea.

Other liver-directed treatments were also considered, but none were judged appropriate. Repeat hepatectomy or surgical debulking was not feasible because the metastases were innumerable and distributed throughout both hepatic lobes (**Figs. 6**, **7**); we judged that neither complete resection nor a sufficient reduction in the VIP-producing tumor burden could be achieved while preserving an adequate remnant liver. Furthermore, the early recurrence after the initial partial hepatectomy indicated that parenchyma-sparing resection would not provide durable control. Transarterial chemoembolization or embolization was not curative. Since residual VIP-producing tumor would inevitably remain, it was judged not to be indicated for a patient with multiple liver metastases from a VIP-producing tumor and life-threatening, refractory WDHA syndrome. Total hepatectomy with LT was therefore the only approach capable of eliminating the entire VIP-producing tumor burden.

With regard to the Milan-NET criteria, the present patient fulfilled the principal requirements: a well-differentiated NET (G2), liver-limited metastases without extrahepatic disease, a hepatic tumor burden below 50% of the liver volume, prior curative resection of the primary tumor drained by the portal system (PPPD), and an observation period of 6 months without extrahepatic progression before transplantation 14-16). Although her age exceeded the age limit generally applied in these criteria, age is generally regarded as a relative rather than an absolute criterion; LDLT was therefore considered justifiable as a rescue therapy for life-threatening, medically refractory WDHA syndrome in a patient whose general condition was otherwise preserved.

Although LT carries substantial risks, transplantation eliminated the source of excessive VIP production and markedly improved the patient’s QOL. After LDLT, the symptoms resolved completely, supporting the therapeutic rationale.

Thus, in carefully selected patients, LDLT may be a valuable therapeutic option for VIPoma by providing both oncological control and symptomatic relief.

## CONCLUSIONS

We report an extremely rare case in which a nonfunctional PNEN acquired the ability to secrete VIP during the clinical course, leading to severe WDHA syndrome. Although medical treatment and surgical debulking failed to achieve adequate symptom control, LDLT for liver-limited disease resulted in the complete resolution of symptoms and favorable outcomes. In selected patients with advanced PNEN, including VIPoma, LT may be a valuable therapeutic option for both oncological control and improvement of QOL.

## DECLARATIONS

### Availability of data and materials

The data supporting the findings of this case are available from the corresponding author upon reasonable request.
